# Evaluation of health outcomes in osteoarthritis patients after total knee replacement: a two-year follow-up

**DOI:** 10.1186/1477-7525-8-87

**Published:** 2010-08-19

**Authors:** Feng Xie, Ngai-Nung Lo, Eleanor M Pullenayegum, Jean-Eric Tarride, Daria J O'Reilly, Ron Goeree, Hin-Peng Lee

**Affiliations:** 1 Programs for Assessment of Technology in Health, St. Joseph's Healthcare Hamilton, Hamilton, L8P 1H1, Canada; 2 Department of Clinical Epidemiology and Biostatistics, Faculty of Health Sciences, McMaster University, Hamilton, L8 S 4L8, Canada; 3 Department of Orthopaedic Surgery, Singapore General Hospital, 169608, Singapore; 4 Centre for Evaluation of Medicine, St. Joseph's Healthcare Hamilton, Hamilton, L8N 1G6, Canada; 5 Centre for Health Services Research, National University of Singapore, Singapore; 6 Department of Community, Occupation, and Family Medicine, Yong Loo Lin School of Medicine, National University of Singapore, 119228, Singapore

## Abstract

**Objectives:**

To quantify the improvement in health outcomes in patients after total knee replacement (TKR).

**Methods:**

This was a two-year non-randomized prospective observational study in knee osteoarthritis (OA) patients undergone TKR. Patients were interviewed one week before, six months after, and two years after surgery using a standardized questionnaire including the SF-36, the Oxford Knee Score (OKS), and the Knee Society Clinical Rating Scale (KSS). A generalized estimating equation (GEE) model was used to estimate the magnitudes of the changes with and without the adjustment of age, ethnicity, BMI, and years with OA.

**Results:**

A total of 298 (at baseline), 176 (at six-months), and 111 (at two-years) eligible patients were included in the analyses. All the scores changed significantly over time, with the exception of SF-36 social functioning, vitality, and mental health. With the adjustment of covariates, the magnitude of changes in these scores was similar to those without the adjustment.

**Conclusions:**

Both general and knee-specific physical functioning had been significantly improved after TKR, while other health domains have not been substantially improved after the surgery.

## Introduction

Osteoarthritis (OA), a chronic degenerative disease, is characterized by pain and physical disability, with knee being the most frequently affected joint[1]. OA is among the most prevalent diseases affecting adults and a major contributor to physical disability, morbidity, and utilization of health care resources worldwide[2-5]. In patients with severe knee OA who have failed conservative treatments (e.g. medications, exercises, and weight loss), total knee replacement (TKR), a surgical option involving replacement of knee joint with artificial components, has been shown to be a highly effective treatment that could result in substantial improvement in physical functioning[6].

It is known that pain, physical functioning, and health-related quality of life (HRQoL) are important outcome measures in OA. Recently there is growing literature that has contributed to the understanding on what could be achieved by TKR[7-10]. Both disease-specific functional measures such as the Western Ontario and McMaster Universities Osteoarthritis Index (WOMAC)[11-14], the Oxford Knee Score (OKS)[15], and the Knee Society Clinical Rating Scale (KSS)[11,16], and generic HRQoL instrument such as the SF-36[11,13,14,16-20] have been used to evaluate the improvement in functioning and quality of life in patients undergone TKR. However, such data are particularly lacking for Asian patients. As prevalence of OA is increasing, TKR is expected to play an important role in reducing pain and improving physical functioning and HRQoL of patients[21]. Thus, there is a pressing need to obtain more empirical evidence on health outcome improvement after TKR in Asian populations.

Therefore, the objective of the present study was to quantify the improvement in health outcomes in Asian patients after TKR.

## Patients and Methods

This was a two-year non-randomized prospective observational study. The institutional review board at the Singapore General Hospital (SGH) had approved this study and patient informed consent forms were collected.

### Patients

A total of 242 patients would be required to detect an effect size of 0.18 using the SF-36[22] with a significance level of 0.05 and the power of 0.8[23]. The inclusion criteria were: (1) patients diagnosed with knee OA based on clinical and radiographic features and received TKR in the SGH between January 1, 2003 and December 31, 2003 (index dates); (2) patients who had not undergone either TKR or other knee surgeries at least six months before the index dates, and (3) patients who had consented to participate in this study. Each patient was interviewed in English by a trained interviewer one week before, six months after, and two years after surgery using a standardized questionnaire including a generic HRQoL instrument (i.e. the SF-36) and two functioning instruments (i.e. the OKS and the KSS). Demographic information for each participating patient was also collected before the surgery.

### Questionnaires

The SF-36, one of the most widely used generic HRQoL instruments worldwide, contains 36 items which measure perceived health in 8 domains, namely, physical functioning, role physical, bodily pain, general health, vitality, social functioning, role emotional, and mental health, with higher scores (range, 0-100) reflecting better perceived health[24].

The KSS consists of two scores, a knee score and a functioning score, both ranging from 0 (worst health or functioning) to 100 (best health or functioning)[25]. The knee score reflects an objective measurement as well as patient-reported pain severity. Fifty of 100 points in the knee score are allocated to pain assessment with 50 representing no pain, while the other 50 points are allocated for a clinical assessment of range of motion, stability, alignment, and muscle power of knee with 50 representing at least 0°-125°of knee flexion with no active lag, no instability, and normal alignment. The function score reflects patient-reported walking distance and stair-climbing and makes deductions for use of a walking aid, with 100 representing unlimited walking distance and normal stair-climbing without use of an aid.

The OKS, a procedure- and joint-specific functioning measure, consists of 12 questions assessing pain and physical disability using a 5-point Likert-type scale, which generates a single score ranging from the worst functional outcome of 0 to the best functional outcome of 100[26].

### Statistical analyses

In order to determine the difference in demographic characteristics of the patients participating in baseline interviews compared to those in post-surgery follow-up interviews, chi-square test and one-way analysis of variance (ANOVA) were used for categorical and continuous variables, respectively. A generalized estimating equation (GEE) model was used to estimate the magnitude of changes in these outcomes over time with and without the adjustment of age, ethnicity, BMI, and the number of years with OA.

The unadjusted marginal model was:

y=α+β1T1+β2T2

and the adjusted marginal model was:

y=α+β1T1+β2T2+β3age+β4ethnicity+β5gender+β6BMI+β7years with OA

Where T1 = 1 if the measurement was taken at six-months and 0 otherwise; T2 = 1 if the measurement was taken at two-years and 0 otherwise; ethnicity = 1 for Chinese and 0 otherwise, and y is the response in question.

The mechanism by which data was missing was investigated by examining which baseline covariates and previous measurements predicted missingness of a given outcome. The only significant predictor was general health at baseline for the missingness at two-years (p = 0.04), and given the number of statistical tests done (40 in all), this is fewer than would be expected by chance alone. It is thus reasonable to conclude that missingness was completely at random and hence does not bias our results. All descriptive analyses were conducted using SAS 9.1 (SAS Institute Inc., Cary, North Carolina, USA), and the remaining analyses were done using R version 2.4.1 (procedures from GEE library). All statistical tests were two-tailed and conducted at 5% significance level.

## Results

The patients' characteristics are shown in Table 1. At baseline, 298 eligible patients participated in the present study with the mean age of 66.8 years. The majority were female (80.4%) with the mean OA duration of 7.8 years and the mean body mass index (BMI, kg/m2) of 27.9. A total of 176 (follow-up rate: 59.0%) and 111 (follow-up rate: 37%) were followed at six-months and two-years after the surgery, respectively. The reasons for the patients lost to follow up were not known. Nevertheless, the demographic characteristics of the patients at six-months and two-years follow-up were comparable to those of the patients at baseline (Table 1).

**Table 1:** Characteristics of the patients

	Pre-surgery	Six-months follow-up	Two-years follow-up
N	**298**	**176**	**111**
Age*, years			
Mean (SD)	66.8(7.6)	66.9(7.8)	66.3(7.9)
			
Female, n (%)	226(80.4)	137(79.7)	84(77.8)
			
Ethnicity, n (%)†			
Chinese	257(92.1)	156(91.2)	97(89.8)
Others	22(7.9)	15(8.7)	11(10.19)
			
Right knee, n (%)	161(54.0)	99(56.3)	64(57.7)
			
Years with OA, mean(SD)	7.8(3.8)	7.7(3.5)	7.7(3.8)
			
BMI (kg/m^2^), mean(SD)	27.9(4.3)	28.1(4.2)	28.2(4.1)
< 25, n (%)	101(34.5)	57(32.8)	33(30.3)
25-29.9, n (%)	116(39.6)	72(41.4)	45(41.3)
> 30, n (%)	76(25.9)	45(25.9)	31(28.4)

TKR=total knee replacement; SD=standard deviation; OA=osteoarthritis;

BMI=body mass index; OKS=Oxford Knee Score.

*Ages were based on pre-surgery values.

†Other ethnicity included Malay, Indian and others.

The observed mean scores of SF-36 physical functioning, role physical, bodily pain, general health, and role emotional, the OKS, the KSS knee and functioning scores changed significantly over time, while the mean scores of SF-36 social functioning, vitality, and mental health did not change significantly (Table 2).

**Table 2:** Mean (standard deviation) health outcome scores of patients before and after surgery*

	Pre-surgery	Six-months follow-up	Two-years follow-up
SF-36			
Physical functioning	32.7(20.2)	55.4(23.4)	59.8(23.6)
Role physical	38.8(40.7)	71.9(41.5)	68.9(42.7)
Bodily pain	41.7(14.3)	47.6(18.0)	40.9(14.0)
General health	56.1(8.9)	56.2(9.0)	52.2(8.3)
Role emotional	81.2(38.6)	96.8(16.2)	93.3(23.8)
Social functioning	52.8(14.0)	54.3(15.6)	51.0(9.7)
Vitality	56.4(12.8)	56.2(13.4)	55.9(11.2)
Mental health	64.7(10.2)	65.9(11.4)	65.5(8.7)
			
Oxford Knee Score	49.1(16.9)	77.7(15.4)	83.1(13.5)
			
Knee Society Clinical Rating Scale			
Knee score	47.5(16.0)	85.0(12.3)	89.1(5.9)
Functioning score	46.2(20.1)	62.4(22.0)	67.3(21.6)

*The GEE does not provide a global p-value to test whether the means were the same across all three time periods, however the p-values comparing 6 months and 12 months vs. pre-op were both < 0.0001.

Table 3 shows the mean changes from the pre-surgery scores predicted by the GEE models. Without the adjustment of demographic characteristics, SF-36 physical functioning score increased by 22.5 at six-months (p < 0.0001) and by 26.7 at two-years (p < 0.0001). Role physical score increased by 32.9 at six-months (p < 0.0001) and 28.7 at two-years (p < 0.0001). Bodily pain score increased by 6.0 at six-months (p = 0.0003), but the change was not significantly at two-years. General health score did not change significantly at six-months and decreased by 4.1 at two-years (p < 0.0001). Role emotional score increased by 15.6 and 12.2 at six-months (p < 0.0001) and two-years (p = 0.0001), respectively. The score increments at six-months were 28.5, 37.5, and 16.2 for the OKS, and the KSS knee and functioning, respectively, while the corresponding increments at two-years were 33.4, 41.3, and 20.9 (all ps < 0.0001).

**Table 3:** Results of the generalized estimating equation model without and with adjustment of demographic characteristics*

Outcome	Unadjusted		Adjusted	
	
	Six-month	Two-year	Six-month	Two-year
**SF-36**				
Physical functioning	22.5 (1.65) < 0.0001	26.7 (2.09) < 0.0001	22.8 (1.95) < 0.0001	27.3 (2.51) < 0.0001
Role physical	32.9 (3.37) < 0.0001	28.7 (4.45) < 0.0001	35.9 (4.00) < 0.0001	26.8 (5.40) < 0.0001
Bodily pain	6.04 (1.46) 0.0003	-0.57 (1.56) 0.7100	4.48 (1.72) 0.0093	-1.41 (1.96) 0.4715
General health	0.12 (0.81) 0.8800	-4.13 (0.90) < 0.0001	0.34 (1.01) 0.7336	-4.23 (1.16) 0.0003
Role emotional	15.6 (2.60) < 0.0001	12.2 (3.20) 0.0001	15.9 (3.37) < 0.0001	12.9 (3.96) 0.0011
Social functioning	1.54 (1.28) 0.2310	-1.52 (1.22) 0.2120	0.81 (1.76) 0.6466	-2.52 (1.72) 0.1431
Vitality	-0.202 (1.21) 0.8670	-0.584 (1.33) 0.0600	-1.08 (1.53) 0.4819	0.15 (1.74) 0.9294
Mental health	1.18 (0.93) 0.2050	0.57 (0.95) 0.5510	2.04 (1.09) 0.0613	-0.07 (1.28) 0.9569
**OKS**	28.5 (1.22) < 0.0001	33.4 (22.6) < 0.0001	28.8 (1.56) < 0.0001	32.4 (1.74) < 0.0001
**KSS**				
Knee	37.5 (1.32) < 0.0001	41.3 (1.55) < 0.0001	37.0 (1.68) < 0.0001	40.4 (2.12) < 0.0001
Functioning	16.2 (1.52) < 0.0001	20.9 (1.90) < 0.0001	15.8 (1.79) < 0.0001	19.4 (2.27) < 0.0001

OKS: Oxford Knee Score; KSS: Knee Society Clinical Rating Scale.

*Numbers are the mean change from pre-surgery with standard error in parenthesis and p value.

With the adjustment of age, gender, ethnicity, BMI, and years with OA, the magnitude of predicted changes in these scores were similar to those without the adjustment. Physical functioning score increased by 22.8 at six-months (p < 0.0001) and 27.3 at two-years (p < 0.0001). The corresponding increments were 35.9 (p < 0.0001) and 26.8 (p < 0.0001) for role physical and 15.9 (p < 0.0001) and 12.9 (p = 0.0011) for role emotional. The score increments at six-months were 28.8, 37.0, and 15.8 for the OKS, and the KSS knee and functioning, respectively, while the corresponding increments at two-years were 32.4, 40.4, and 19.4 (all ps < 0.0001).

## Discussion

In this two-year prospective study, statistically significant improvements were observed in the generic SF-36 physical functioning, role physical, and role emotional domains and in the two disease-specific instruments. After the adjustment of covariates including age, gender, ethnicity, BMI, and years with OA, the results were similar. The magnitude of the improvements also exceeded the minimally important difference reported for the SF-36[22]. TKR, as an effective surgery option for severe OA patients, can substantially improve both general physical functioning (as measured by the generic SF-36) and knee-specific physical functioning, and reduce knee-related pain (as measured by the OKS and the KSS). However, no significant improvement in other aspects of health (e.g., mental and social health) or general health has been observed.

The improvement in knee functioning and substantial reduction in knee pain as measured by the OKS and the KSS were consistent with previous studies[13-17], as was the physical functioning and role physical measured by the SF-36[13,14,17-20]. Surprisingly no significant change in SF-36 bodily pain score at both six-months and two-years was observed. This finding was different from some published studies[9,10,13,14,17-20,22], which reported that SF-36 bodily pain had also been reduced significantly after TKR. Though it is not clear about the true answer to this contrast finding, there are several possible explanations. First is the presence of comorbid back pain in this patient population. SF-36 bodily pain domain was designed for general bodily pain (e.g. back pain) as opposed to knee pain. Veerapen *et al*., found that back pain was more common than knee joint pain in Asian populations[27] and back pain was reported as a significant factor influencing post-TKR SF-36 bodily pain, vitality, and mental health scores[9]. This might be a possible reason why SF-36 bodily pain had demonstrated minimal improvement after surgery if back pain was a common comorbid condition for this patient population. However, the prevalence of back pain was not captured in the present study. It is thus suggested that the information be collected in future studies. Second is the difference in patient characteristics. The patients enrolled in previous studies were either younger[10] or older[9,22], and with higher BMI[9,10,22]. Bugala-Szpak et al., found that BMI, rather than sex and age, had a significantly influence on post-TKR quality of life scores[17]. A large study is necessary to confirm this finding. Thirdly and importantly, ethnic differences in pain perception between Asian and Western populations might contribute to this discrepancy. Thus caution should be exercised when generalizing the results to other ethnic groups.

Social and mental health as measured by the SF-36 remained unchanged or even a little worse after surgery. Singer *et al*., suggested that there might be a strong psychological adjustment or adaptation to physical disability in the elderly[28]. Nevertheless, patients' social and mental health was still less satisfactory compared to the same age group of Asian populations[29]. Ayers *et al*., reported that poorer pre-TKR mental health might have a negative impact on the improvement of post-TKR physical functioning[30]. Escobar *et al*., also found that pre-TKR mental health was a significant factor predicting post-TKR physical functioning[9]. Some studies have demonstrated that social support might play an important role in moderating the effects of pain, physical disability, and depression in patients with OA[31-36]. All these evidence may suggest that providing social and mental support to this patient population could be an important way of improving their quality of life in the long term.

The study had higher drop-out rates in following up the patients. A sensitivity analysis was conducted by calculating the mean of the outcome measures at each time point using all available measurements and comparing with those using completers only, and this made very little difference. General health of patients was worse at two-years than that at baseline. General health is also the only significant predictor for the missingness at two-years. This finding was not surprising as more than 80% of the patients were aged over 60 and 40% over 70. Although these patients might be seen in other departments later on, it would be difficult for them to come back to the orthopedic department to complete an additional examination two years after the surgery unless knee OA is getting worse.

In conclusion, both general and knee-specific physical functioning had been significantly improved after TKR, while other health domains remained unchanged after the surgery.

## Competing interests

The authors declare that they have no competing interests.

## Authors' contributions

FX designed the study, participated in data collection, data analysis, results interpretation and took the lead on drafting the manuscript and subsequent revisions. NNL participated in data collection and provided clinical expertise. EMP participated in the data analysis and results interpretation, as well as contributing to writing the manuscript. JET, DJO and RG participated in results interpretation and also contributed to writing the manuscript. HPL participated in the data collection and results interpretation. All authors read and approved the final version of the manuscript.
